# Analysis of the Diversity of Megachilidae Bees on the Northern Subplateau of the Iberian Peninsula

**DOI:** 10.1673/031.010.20701

**Published:** 2010-12-08

**Authors:** Maríía Joséé Dardóón, Féélix Torres, Josep Daniel Asíís

**Affiliations:** Departamento de Biologíía Animal, Ecologíía, Parasitologíía y Edafologíía. Universidad de Salamanca. Campus Miguel de Unamuno s/n. E-37071 Salamanca, Españña

**Keywords:** complementarity, landscape unit, Malaise trap, taxonomic distinctness measures

## Abstract

In the western Mediterranean, 772 species of bees in the family Megachilidae have been reported. Special emphasis has been placed on the Iberian Peninsula, where to date 218 species are known. However, few intensive studies providing information about communities of Megachilidae have been carried out. Two earlier works cite 70 species; almost one third of those known on the Peninsula. With an aim of gaining insight into the structure of the communities of Megachilidae and the factors influencing them, an analysis was made of the *alpha* and *beta* diversity of different localities in the northern subplateau. Malaise traps (black and white) were used, and 559 specimens belonging to 55 species were identified of which most exhibited a nest-holder-type nesting habit. Abundance and richness were higher for white traps, although a considerable degree of complementarity was observed with the black traps. In the study zone, diversity can be considered medium-high with a phylogenetic diversity corresponding to stable populations. Regarding the composition of the Megachilidae communities, the influence of the landscape structure, of the microhabitat, and of the colour of the trap used to collect the specimens was detected. The following are recommended: (1) the use of both black and white traps, since they show high complementarity and offer different information about community structure, (2) homogenization of the samples in comparisons among communities, owing to the influence of the color of the trap, which masks the importance of ecological factors in community structuring, and (3) the collection of samples from at least two years previous, in view of the elevated “replacement” of species observed with species richness estimators.

## Introduction

The conservation of biological diversity is a priority issue owing to its accelerated loss, with irreversible effects on the disappearance of taxonomic groups of which nothing is known with regards to either presence or their roles in ecosystems (Gaston 1991; Mayr 1992; Wilson 1994). Insects are one of the groups with the best projections for the description of new species (Erwin 1982; Primack and Ros 2002), such that conservation projects should include this group in inventories and should perform analyses of their diversity (Wilson 1987; Hawksworth and Ritchie 1993; New 1999).

Currently, there are many statistical methods available that allow diversity to be analyzed in terms of biology, ecology, and taxonomy. Analyses of *alpha* diversity are very common in studies addressing diversity, evaluating the species richness of a community, and *beta-*diversity analyses allow the composition of species from different communities to be compared. Normally, measurements of similarity and complementarity are used. Recently, however, the use of taxonomic distinctness measures has been implemented (Clarke and Warwick 2001a) in insect communities (Anu and Sabu 2006; Bañños-Picóón et al. 2009). Such indices enable contrasts to be made between real data, thus evaluating the structure of the population with respect to the phylogenetic patterns of the group within a given area.

Bees, in particular the Megachilidae, constitute an important group as regards the sustainability of ecosystems because they are involved in the pollination of a large number of plants and exhibit a great diversity of forms, sizes, and behavioral traits (O'Toole and Raw 1991; Michener 2000). In the case of the Iberian Peninsula, 218 species of Megachilidae have been reported, although no exhaustive studies have been performed and the few works that have been published offer only lists of the fauna and provide little information about community structure.

One of the most effective ways of capturing flying insects is using Malaise traps, especially for the collection of Diptera and Hymenoptera which account for 90% of captures (Matthews and Matthews 1983; Tereshkin and Shyakhtenok 1989). Malaise traps allow quantitative data to be obtained with homogeneous samples and allow these to be interpreted as frequencies in abundance analyses of the individuals captured. They also provide information about community diversity, population sizes, temporal distribution, and different aspects of insect ecology (Masner and Goulet 1981; Kuhlmann 1994). Townes (1972) reported that a difference in trap colour may lead to differences in insect capture and in different works it has been demonstrated that, at least for particular groups, trap color is fairly important (Krč?mar and Durbeššić? 1997; Gonzáález et al. 1999; Campbell and Hanulla 2007). Nevertheless, to date the influence of trap color on the capture of Megachilidae remains unknown.

In the present work Megachilidae diversity was analyzed by using white and black Malaise traps at two localities on the northern subplateau of the Iberian Peninsula over two years. The values recorded with those of other authors in the same or other landscape units were also compared, also using Malaise traps. The goals of the present work were: 1) to evaluate the possible influence of the color of the Malaise trap on the perception of communities of Megachilidae in terms of richness, heterogeneity, and guild composition, 2) to determine whether the effect of the traps could be stronger than that of landscape at a broad scale (Landscape Units) by comparing the samples obtained with Malaise traps corresponding to different landscape units, and 3) to analyze the species accumulation curves, in order to establish a duration of the sampling period that would ensure a suitable representation of existing bee diversity.

## Methodology

### Study area

**Figure 1.**
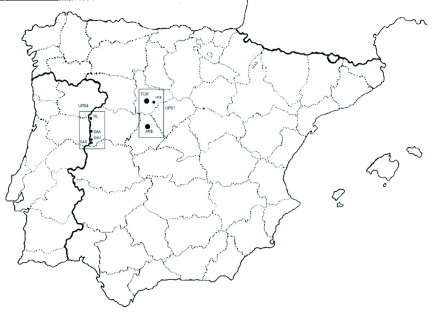
Sampled localities in the northern subplateau of the Iberian Peninsula. TOR, Tordesillas; ARE, Aréévalo; VCE, Viana de Cega; VIL, Villarino de los Aires-Huerto; SAU, Saucelle; SAA, Salto de Aldeáávila; SAS, Salto de Saucelle. High quality figures are available online.

The Megachilidae fauna was studied at two sites (Aréévalo and Tordesillas, Figure 1) on the northern subplateau of the Iberian Peninsula. Both are within the Low Supramediterranean (Rivas-Martíínez 2002) bioclimate, characterized by low levels of precipitation (0 –36 mm per annum) and medium-high temperatures (from 2.2 °°C in winter and up to 26 °°C in summer). At Aréévalo, sands and clays predominate in the sediments with calcic Luvisols and Arenosols as characteristic soil units of the region, while at Tordesillas, the geological materials comprise clays and clayey marls. At both localities, the soil units correspond to Calcisols, Regosols, Luvisols, and Vertisols with a pine cover over sandy soils (Garcíía et al. 1985; FAO 2006).

From the phytogeographical and geobotanical points of view, both localities are in the Mediterranean Peninsular region proper, and to a large extent they have been denuded of the autochthonous forest cover owing to the increased land use for agricultural purposes (Garcíía et al. 1993; Sanz et al. 2003). Their climax vegetation corresponds to the Low Supramediterranean bioclimate and they belong to Landscape Unit 51: Open countryside of the Northern Meseta (Sanz et al. 2003). Their surroundings are planted in cereal crops, mainly barley, *Hordeum vulgare* L. (Poales: Poaceae). The communities representing this type of vegetation, such as *Stellarietea mediae*, are partly displaced to other nearby biotopes, such as ditches and dividers.

### Data collection

To collect specimens, 15 commercial Malaise traps (light-weight Townes (1972) model) were used with a fine mesh (0.1 mm) in black and white (B & S Entomological Services, www.entomology.org.uk/). The traps were deployed at the two localities studied in 2005 and 2006; they were placed in open areas with shrubs in the transition between pine copses and land devoted to cereal crops. Traps were exposed to full sunshine and were oriented to the southwest. The 9 traps used at Tordesillas were in uninterrupted use from 15 April to 30 September, while the 6 placed at Aréévalo were used from 1 May to 15 October of both years (the exact location of the traps is presented in Table 1). Samples were collected at intervals of 15 days and the specimens obtained were preserved in 70% ethanol until their identification, this being performed to the species level following the taxonomic ordering adopted in Ornosa et al. (2006, 2007, 2008).

**Table 1.**
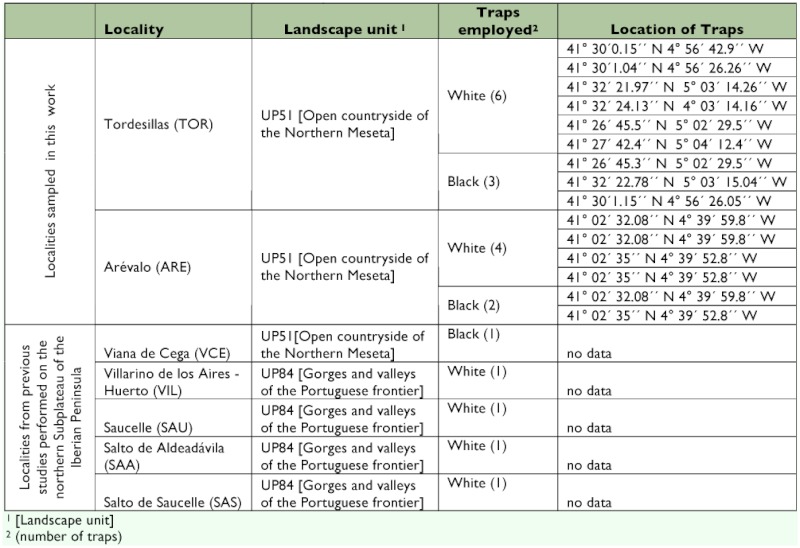
Localities of the northern Subplateau analyzed.

A matrix with data on abundance, collecting period, taxonomic description, and the nesting habits of the specimens identified was constructed. For the analysis of Average Taxonomic Distinctness (Delta+) and Variation in Taxonomic Distinctness (Lambda+) a master list was compiled (Clarke and Warwick 2001a), which contained all the species of Megachilidae cited for the Iberian Peninsula, including all the supraspecific taxa up to family level (see Ornosa et al. 2006, 2007, 2008) and the different nesting habits.

To study the diversity components, two main types of analyses were performed: (1) an analysis of *alpha* diversity using species richness indices, richness estimators (non-parametric and asymptotic), proportional abundance indices, and indices of taxonomic diversity; and (2) an analysis of *beta* diversity, for which the data published for another 5 localities on the northern subplateau was included and sampled with the same method (Table 1). The localities belonged to the Arribes del Duero region (VIL, SAA, SAU, SAS) on the Spanish-Portuguese border with one-year samples between 1995 and 1998 (Gonzáález 2002), and the locality of Viana de Cega (VCE) was sampled over one year between 1993 and 1994 (Gonzáález et al. 1999).

### Statistical analyses

To analyze the communities cluster analysis, ANOSIM (Analysis of Similarities) (Clarke and Green, 1988), and multidimensional scaling (MDS) (Kruskal and Wish 1978) were used, these also calculated the complementarity index from the expression proposed by Colwell and Coddington (1994). MDS was constructed based on square-root transformed abundances and a Bray-Curtis similarity matrix. A species analysis by nesting guilds was also performed establishing five categories: 1) kleptoparasitoids, 2) nest holder, 3) mason + nest holder, 4) terricolous + nest holder, and 5) xylicolous + nest holder. Nest holders are species that occupy pre-existing cavities to establish their nests. For the analyses, PRIMER v5 (diversity indices, cluster analysis, taxonomic distinctness measures) (PRIMER-E Ltd, www.primer-e.com). XLSTAT 2008 (χ?^2^, non-parametric analyses) (Addinsoft TM), and Estimates 7.5.2 (estimation of species richness) statistical packages were used. To elaborate the cluster analysis, the Bray-Curtis index (Bray and Curtis 1957) was employed, using simple joining as the clustering method and previously removing the influence of very rare species (those with abundances of less than 2%) and reducing the weight of the dominant species (by square-root transformation of the abundances).

## Results

### Analysis of *alpha* diversity

A total of 559 specimens were identified, corresponding to 55 species of Megachilidae (Table 2). Forty-five species were collected at Tordesillas and 28 at Aréévalo; 10 of the 28 at Aréévalo were found exclusively at this location. To make comparisons between the two localities, only the data obtained between May and September were included. The summer months afforded greater abundance and richness, both being higher at Tordesillas during June (Table 3) as is characteristic of heliophilous insects.

Of all the individuals collected, 24.5% were male and 75.5% female (sex ratio 1:3). Females were captured for 95% of the species collected, while males were only represented in 40% of the species. The localities of Aréévalo and Tordesillas had similar abundances despite the difference in the number of traps used at each site (Tables 1, 3). Richness was higher at Tordesillas, with the presence of 82% of the species identified, whereas at Aréévalo, a higher number of exclusive species was captured: approximately 18%. From the point of view of the fauna, the presence of *Hoplitis lepeletieri* Péérez, *Hoplitis papaveris* Latreille, and *Hoplosmia ligurica* Morawitz, known only in the north-eastern part of the Iberian Peninsula (Pyrenees, Catalonia, and Barcelona, respectively), is interesting because their distribution is considerably expanded to the west part of Spain. Additionally, the presence of a new species of *Chelostoma* was detected; this is currently undergoing descriptive study.

**Table 2.**
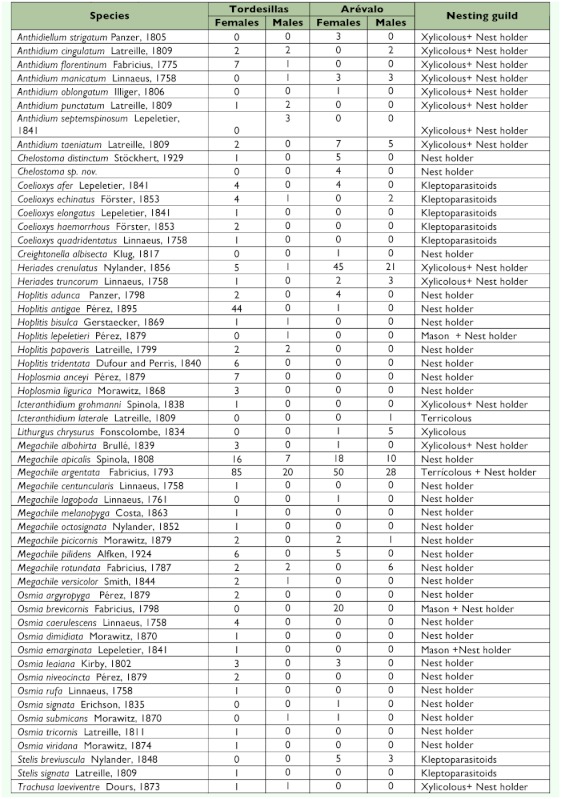
List of Megachilidae species at the localities studied

Analysis of the abundances obtained during the monthly samplings reflects the existence of differences between the two localities studied (χ?^2^_4_=26.05, p<0.0001), even though both of them belong to the same Landscape Unit (Table 3). At Tordesillas, abundances in June were higher than expected (when *Hoplitis antigae* Péérez, and *Megachile argentata* Fabricius were especially abundant) and lower in September. At Aréévalo, the June abundances were lower than expected, while for September they were higher, probably influenced by the strong presence of *M. argentata.*

The differences observed in richness between the two localities during the different months of collection (Table 3) were not significant (Kolmogorov-Smirnov test: D= 0.800, p= 0.079), although it should be noted that the value of the associated probability, close to 0.05, is submarginal. However, the communities were clearly different in regards to composition as revealed by the value of the complementarity index, which was 67.3% between the localities. The dominant species were *Megachile argentata, Megachile apicalis* Spinola, *Hoplitis antigae*, and *Heriades crenulatus* Nylander, representing 60% of all the specimens collected during the period. The two species of *Megachile* showed a broad temporal distribution, from May to September, during which *H. antigae* and *H. crenulatus* exhibited clear demographic bursts in certain months (June and July, respectively).

**Table 3.**
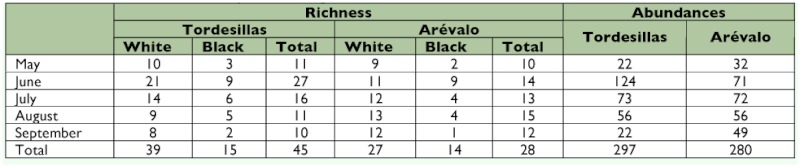
Richness and abundance of species of Megachilidae collected at the localities studied.

**Table 4.**
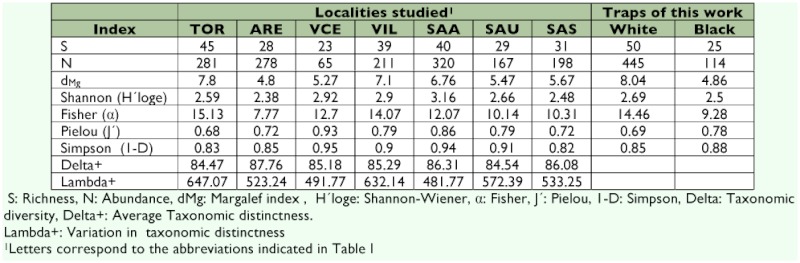
Conventional and taxonomic diversity indices of Megachilidae communities for the localities of the northern Subplateau of the Iberian Peninsula.

The different diversity indices calculated afforded high values and revealed high diversity and low dominance at both localities studied (Table 4). Of the diversity values calculated according to trap color, the high efficiency of the white Malaise traps in the capture of a higher number of species and individuals in comparison with the black ones was striking.

**Figure 2.**
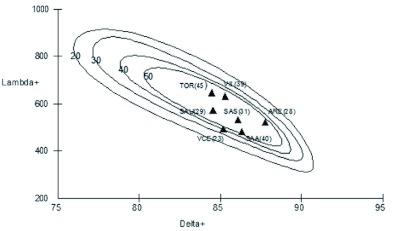
Probability limits (5%) for the Delta+ and Lambda+ values, represented for different sample sizes (between 20 and 50 species) obtained by simulation (1000 restarts) from the master list of the Megachilidae of the Iberian peninsula. The values obtained for the seven communities analyzed are shown. Letters correspond to the abbreviations indicated in Table 1. High quality figures are available online.

Average Taxonomic Distinctness (Delta+) at the two localities indicated that the community of Megachilidae at Aréévalo shows a greater mean taxonomic distance (i.e. it occupies a greater ““average phylogenetic space””) than that seen at Tordesillas (Table 4), and was the one that also had the highest value of the localities sampled on the northern subplateau. In any case, the Delta+ and Lambda+ values did not vary substantially for either of the localities from those obtained by simulation from the master list corresponding to the Iberian Peninsula (Figure 2).

### Species diversity by nesting guilds

Most of the species collected at the two study localities were classified as nest holders (Table 2). This type of behavior confers them greater availability with regards to nesting sites compared with bees that have to construct the gallery of their nests, for which they must invest more time and effort. Comparison of the abundances of both localities, according to nesting guilds, revealed significant differences (χ?^2^_4_=73.387, p< 0.0001). In Tordesillas nest holders were more abundant, whereas in Aréévalo the mason + nest holder and xylicolous + nest holder (Table 5) types were comparatively dominant. The differences could be due to a greater availability of the resources needed to build their nests at Aréévalo such as pithy stems, leaves, and clayey soils.

**Table 5.**
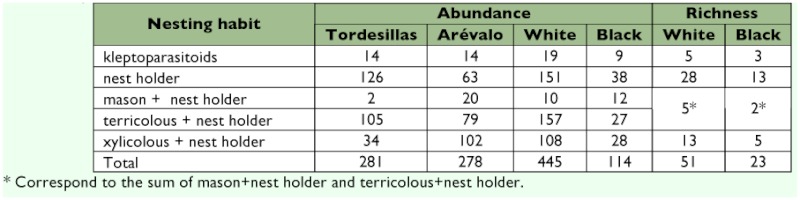
Species abundance and richness by nesting guilds as a function of trap colour.

Comparison of richness between both traps, according to nesting guilds, did not reveal differences (χ?^2^_3_= 0.274, p= 0.965) (i.e. the proportional richness by guilds was the similar). Although globally, as reported above, the richness estimated with the white traps was higher (Table 5). Nevertheless, upon analyzing the abundances of the different nesting guilds as a function of trap color, significant differences were observed between the white and the black traps (χ?^2^_4_= 21.905, p< 0.001). The guilds kleptoparasitoids and mason + nest holder were those with the lowest abundances in both types of trap. The greatest differences attributable to color appeared in the mason + nest holder guild, which was over-represented in the black traps. This suggests a better efficiency of those traps for the capture of species of this guild, which would give rise to different ““perceptions”” of the communities as a function of the color of the trap used.

### Analysis of *beta* diversity

**Figure 3.**
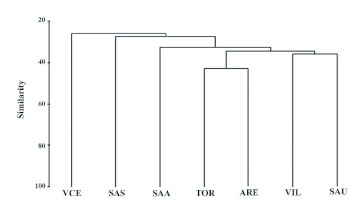
Dendrogram of the cluster analysis of the seven communities of Megachilidae of the Iberian Peninsula studied (Bray-Curtis similarity index; clustering method: single linkage). Letters correspond to the abbreviations indicated in Table 1. High quality figures are available online.

The localities of Tordesillas and Aréévalo showed a similarity that did not reach 50% and a complementarity close to 70% (Table 6). Also, the results of the similarity analyses of the different communities of Megachilidae on the northern subplateau for which data obtained with Malaise traps were available revealed that the communities analyzed had low similarity and high complementarity (> 0.53). The community at Viana de Cega was the one showing the lowest similarity with the rest of the localities analyzed (slightly above 20%) (Figure 3).

## Discussion

### Analysis of *alpha* diversity

White traps collected 80% of the individuals, while only 20% were collected with the black ones. This points to the greater effectiveness of white Malaise traps in capturing specimens of Megachilidae (Kolmogorov-Smirnov D=1.000, p=0.008), which is the general case of bees and other taxa (Gonzáález et al. 1999; Gayubo et al. 2000; Gonzáález 2002).

**Table 6.**
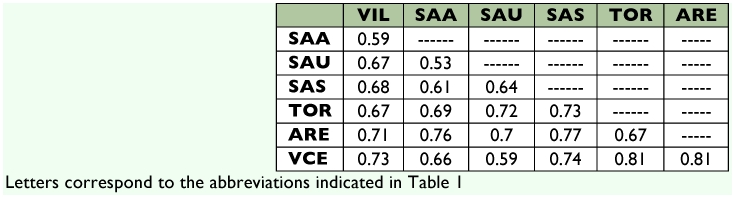
Values of the complementarity index of the seven communities of Megachilidae on the northern Subplateau of the Iberian Peninsula analyzed with Malaise traps.

The abundance of specimens collected was greater from June to August (Table 3). However, the analyses revealed that trap color did not favor the capture of bees in any given month (χ?^2^_4_= 7.936, p= 0.094), as was the case of species richness. In any case, it should be noted that the black traps captured 5 exclusive species with a single individual each, highlighting the importance of their use in fauna studies on bees. This is supported by the complementarity (65.5%) between the black traps and the white ones.

The sex ratio of the Megachilidae studied here is contrary to what was expected for solitary bees, in which the ratios tend to range from 1:1 to 10:1 with males being more numerous than females (Torres 1992; Gonzáález et al. 1999). This may be due to the fact that Malaise traps capture specimens on the wing, such that individuals that move more frequently and over greater distances increase the probability of their being captured. Females in search of nesting sites and food for provisioning their cells usually travel farther than males (which could patrol in different or more restricted microhabitats in search of mates), such that the probability of their being captured is greater. So trap placement could have underestimated male abundances.

### Indices of conventional diversity

The moderately high diversity values (medium-high) obtained are undoubtedly related to the place where the traps were set up: the transition between the pine copses and land devoted to cereal crops that provide food for the bees. In comparison with cultivated land and forests, these zones are considered to have the greatest diversity of bees (Duelli et al. 1999; Marshall et al. 2006) since they harbour phanerogams with a herbaceous habit, the basic source of nutrition for the bees.

On comparing the two localities addressed here, it may be seen that Tordesillas has a greater richness of species collected: Tordesillas accounted for 80% of the total of species identified as compared with 51% found for Aréévalo, even though the abundances were fairly similar at both localities (Table 3). This is reflected in the values obtained with the ““richness”” indices (d_Mg_, H', and α?) that were much higher at Tordesillas. Nevertheless, the community at Tordesillas shows a greater dominance than that of Aréévalo, as seen in the lower values of the Pielou and Simpson indices (J' and 1-D, respectively) for Tordesillas (Table 3), and these would reflect less equitable distributions of the species abundances of the community.

On comparing the diversity indices of all the localities sampled on the northern subplateau (Table 4), the high value of the richness component reached by the Tordesillas community is striking, with d_Mg_ and Fisher α? indices higher than those recorded in the other communities. This value is similar to that obtained for Villarino a locality for which high richness values have been reported for other groups of hymenopterans (Gayubo et al. 2000). However, the heterogeneity component obtained for the pine-stand samples (TOR and ARE) (assessed with the J' and 1-D indices) is relatively low in comparison with those found for the Arribes del Duero samples (VIL, SAA, SAU, SAS) or that of Viana de Cega (VCE) revealing slightly less balanced communities in which a few species dominate.

**Figure 4.**
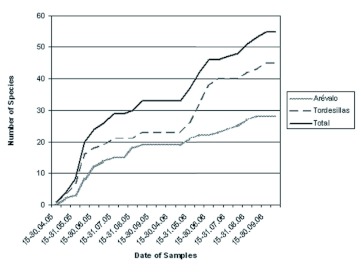
Species accumulation curves for Megachilidae in the two localities under study (Tordesillas and Aréévalo) during the two-year period. High quality figures are available online.

The species richness recorded along the two years of study reflects considerable increases during May and June in both study years. In these months 60% of all species obtained were collected. On estimating the species richness of the Megachilidae community using the Michaelis-Menten function (Michaelis and Menten, 1913), an estimated richness of 79 species was obtained such that the number of species collected in this work represents 76% of the total expected (according to Jiméénez-Valverde and Hortal (2003), proportions above 70% can be considered reasonable estimations for entomological samples). However, on assessing the richness obtained for each of the years separately, the percentage of species found (33 species in 2005, and 51 in 2006) as compared with those estimated is lower (64.7% in 2005 and 59.2% in 2006) (Figure 4), pointing to the need to perform long-term studies in which at least two years of sampling are used.

The white Malaise traps collected a higher number of specimens than the black ones (455 vs. 114). This greater abundance probably accounts for the differences reflected by the richness indices (d_Mg_, Shannon-Wiener and Fisher a), strongly influenced by the sample size (Moreno 2001; Magurran 2003). By contrast, the dominance indices show the samples obtained with the black traps to be more balanced. These observations provide new evidence of the bias introduced in the sampling by trap colour and of the limitations of the results obtained using only this capture method (Townes 1972; Krč?mar and Durbeššić? 1997; Campbell and Hanulla 2007). For studies addressing species inventories where richness is essential, white traps are recommended since they provide higher abundances and richnesses; in contrast, the use of black traps is complementary and, for the analysis of population structures, seems to be indispensable.

### Taxonomic distinctness measures

Average taxonomic distinctness is greater at Aréévalo, this being due to the fact that although the number of species was much higher at Tordesillas and Villarino (45 and 39 species, respectively, as compared with the 28 captured at Aréévalo). The number of genera present at Aréévalo is the same as that of Villarino and higher than that found at Tordesillas (12 vs. 11) with 4 tribes being represented (3 at Tordesillas). Also, the Lambda+ value is higher at Tordesillas and Villarino, pointing to a greater ““inequality”” in the distribution of species in the higher taxonomic categories: some genera represented by many species as compared with others with one or only a few species. This situation contrasts with that observed at Viana de Cega where most of the genera include one or two species (only one genus, *Osmia*, is represented by more than 3 species). In any case, it should be noted that the communities analyzed show values that do not depart significantly from those expected considering the phylogenetic relationships shown by the Megachilidae species present on the Iberian Peninsula. This suggests that the communities are reasonably stable and that they are not being subjected to important disturbances (Clarke and Warwick 2001b).

Note should be taken of the relationship between Delta+ and Lambda+ observed in the simulation, which would reflect a negative correlation between both variables (evident in the slope observed in the simulation ellipses (Figure 2)). Although Clarke and Warwick (2001b) failed to observe a relationship between either variable in their study conducted on the nematode fauna of Great Britain, they did suggest that this relationship could depend on the group and region studied and hence that it can exist. Thus, although the use of these indices is not very widespread in studies addressing insects, an earlier analysis performed with ants also revealed a negative correlation (Anu and Sabu 2006), whereas another work on spheciform wasps reported a positive correlation (Bañños-Picóón et al. 2009). Considering that in the present work the simulation was carried out using the master list of the Megachilidae species of the Iberian Peninsula, the observed correlations would constitute a reflection of the taxonomic structure of that species. Accordingly within this framework the ““empirical”” correlation found, even though it is only based on seven communities (ARE, TOR, SAA, SAU, SAS, VIL, and VCE), would be the expected one for this group in this study zone, and the differences observed between these localities cannot be attributed to external pressure; instead they would be inherent to the distribution of Iberian species of Megachilidae in their upper taxonomic categories and their cladogenesis patterns.

### Analysis of *beta* diversity

Despite their proximity and the fact that they belong to the same landscape unit, Tordesillas and Aréévalo show a complementarity of almost 70%. Different factors such as the fact that the distribution of many terrestrial invertebrates is patchy, that such distribution is strongly influenced by seasonality and that it reveals many species with restricted distributions and populations subjected to fluctuations in abundance, could explain the relatively high complementarity values found in the present study and that appear to be common to other insect communities (Colwell and Coddington 1994; Novotny and Basset 2000; Longino et al. 2002).

Part of the elevated complementarity observed between Viana de Cega, on one hand, and Aréévalo and Tordesillas, on the other, could be due to the bias introduced by trap color, since at Viana de Cega only a black trap was used whereas at the other two localities white traps were also employed. As mentioned above, the complementarity between white traps and black ones (more than 65%) is evidence that the capture achieved with both models is not the same: also, the analysis of abundances by nesting guilds reveals an effect of trap color on captures.

The similarity analysis (Figure 3) shows that Tordesillas and Aréévalo are the localities with the greatest similarity, although this does not reach 50%. Moreover, the Megachilidae community present at Viana de Cega is the one showing the lowest similarity to the rest (only around 20%). Since Viana de Cega has the environmental and landscape characteristics closest to those seen at Tordesillas and Aréévalo (because all three localities belong to UP51 whereas those of the Arribes del Duero (Aldeadáávila -SAA-, Saucelle -SAS, SAU-, and Villarino -VIL-)) lie within UP84 (Sanz et al. 2003), it would be logical to expect greater similarity among those three communities. Nevertheless, the fact that a black trap was used for sampling at Viana de Cega (in the other localities white traps were also used) probably introduced a bias in the sample that would mask the assumed greater similarities due to ecological factors (Figure 3).

**Figure 5.**
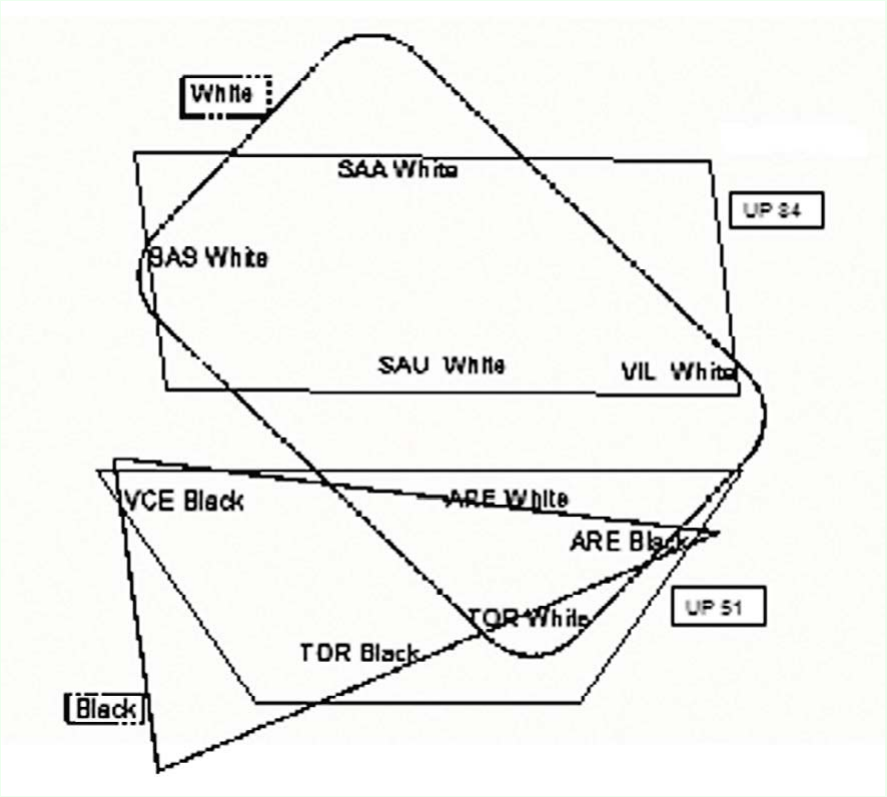
Multidimensional scaling (MDS) of communities of Megachilidae of the northern Subplateau of the Iberian Peninsula analyzed, obtained with traps of different color (Bray-Curtis similarity index) (- - - - colour of trap (black vs. white)) and Landscape Unit (UP51 vs UP84). High quality figures are available online.

To analyse the influence of trap colour and locality separately, a multidimensional scaling (MDS) was performed, dividing the samples as a function of those variables (color and locality). The plot obtained in two dimensions (with a stress value of 0.15) is shown in Figure 5 and it reveals a greater influence of the ecological factor (landscape unit) on the communities. In fact, on performing ANOSIM with the localities grouped in their corresponding landscape units, those belonging to UP51 (Aréévalo, Tordesillas, and Viana de Cega) were significantly separated from those included in the UP84 unit (Aldeadáávila, Saucelle, and Villarino) (ANOSIM, R=0.338; p<0.05). However, when trap color was included as a factor the differences among the groups were not statistically significant (ANOSIM, R=0.173, P=0.143).

The structure of the landscape at a broad scale (Landscape Units) has a strong effect on the composition of the communities of Megachilidae, although the high complementarity values observed, even between localities belonging to the same Landscape Unit, suggest an important influence of the microhabitat on the composition of those communities. Additionally, the influence exerted by trap color, which masks the importance of the landscape structure, makes it necessary to homogenize the samples in comparative studies among communities. In the study of communities of Megachilidae the following are therefore advisable: 1) the use of both black and white traps because they show a noteworthy complementarity and afford different information about community structure; 2) homogenization of the samples in comparisons among communities in view of the effect of trap colour and the fact that it masks the importance of ecological factors in community structuring; and 3) sampling with a duration of at least two annual periods owing to the high ““replacement”” of species observed from the estimators of species richness.
